# Chemosensory protein 4 is required for *Bradysia odoriphaga* to be olfactory attracted to sulfur compounds released from Chinese chives

**DOI:** 10.3389/fphys.2022.989601

**Published:** 2022-09-27

**Authors:** Yuting Yang, Dengke Hua, Jiaqi Zhu, Fu Wang, Youjun Zhang

**Affiliations:** ^1^ Hubei Engineering Technology Center for Pest Forewarning and Management, Institute of Insect Sciences, Yangtze University, Jingzhou, Hubei, China; ^2^ Institute of Agricultural Quality Standards and Testing Technology Research, Hubei Academy of Agricultural Sciences/Hubei Key Laboratory of Nutritional Quality and Safety of Agro Products, Wuhan, Hubei, China; ^3^ Department of Plant Protection, Institute of Vegetables and Flowers, Chinese Academy of Agricultural Sciences, Beijing, China

**Keywords:** Bradysia odoriphaga, chemosensory protein 4, competitive binding assays, RNAi, Y-tube olfaction assay

## Abstract

*Bradysia odoriphaga* (Diptera: Sciaridae) is a serious pest of Chinese chives cultivated in China. Chemosensory proteins (CSPs) are important components of insect olfactory systems that capture and bind environmental semiochemicals which are then transported to olfactory receptors. Despite their importance, the mechanism of olfaction and related behavioral processes in *B*. *odoriphaga* have not been characterized. Here, we found that *BodoCSP4* has an important olfactory function. RT-qPCR indicated that *BodoCSP4* expression was highest in the heads (antennae removed) of adult males, followed by the antennae of adult males. Competitive binding assays with 33 ligands indicated that BodoCSP4 binds well with methyl allyl disulfide, diallyl disulfide, and n-heptadecane; the corresponding dissolution constants (K_i_) were as high as 5.71, 5.71, and 6.85 μM, respectively. 3D-structural and molecular docking indicated that BodoCSP4 has five α-helices and surrounds the ligand with certain hydrophobic residues including Leu60, Leu63, Leu64, Ala67, Val28, Ile30, Ile33, Leu34, and Val86, suggesting these residues help BodoCSP4 bind to ligands. Silencing of *BodoCSP4* significantly decreased the attraction of *B. odoriphaga* males to diallyl disulfide and n-heptadecane but not to methyl allyl disulfide in Y-tube olfaction assays. These results increase our understanding of how *BodoCSP4* contributes to host and female localization by *B. odoriphaga* males.

## Introduction

Insects depend on their olfactory systems to perceive chemical signals including host plant volatiles, sex pheromones, and alarm pheromones (Visser, 1986; Hansson, 2002; French et al., 2015). In insect olfactory systems, chemosensory proteins (CSPs) participate in the transmission of semiochemicals through the sensillar lymph fluid to the olfactory receptors (ORs) (Pelosi et al., 2005; Oliveira et al., 2018), and then transduce electrical signals in order to activate physiological functions and regulate insect behavioral responses (Pelosi et al., 2006; Antony et al., 2016). Therefore, insect CSPs have an indispensable role in the recognition of semiochemicals and help ensure that insects make timely and effective behavioral responses to such chemical signals (Pelosi et al., 2018).

Many studies have shown that insect CSPs are found in multiple tissues, suggesting that CSPs have additionally functions to chemosensation (Picimbon, 2003). In addition to being involved in host volatile recognition (Younas et al., 2022), CSPs are also involved in development (Maleszka et al., 2007), reproduction (Cheng et al., 2015; Zeng et al., 2020), circadian cycle regulation (Claridge-Chang et al., 2001), leg regeneration (Kitabayashi et al., 1998), insecticide resistance (Xu et al., 2022), vision (Zhu et al., 2016), and physiological shifts such as transition from the solitary to gregarious phase of *Locusta migratoria* (Chen et al., 2010; Guo et al., 2011). Therefore, studying how insect CSPs function will help to enrich our understanding of the potential roles of CSPs, and advance the development of comprehensive control methods using insect CSPs as molecular targets.

The Gnat *Bradysia odoriphaga* is a pest of both vegetables and mushrooms and is especially damaging to Chinese chives cultivated in Asian countries (Feng and Zheng, 1987). Feeding by *B. odoriphaga* larvae reduces the growth, development, and edibility of Chinese chives (Li et al., 2014). This pest is difficult to control because of its high fecundity and the soil-borne habitat of the larvae (Dang et al., 2001; Ma et al., 2013). At present, *B. odoriphaga* is mainly controlled via the application of insecticides, but overuse of chemical pesticides results in serious problems including insecticide resistance, environmental pollution, pesticide residues on host plants, and negative effects on human health (Yang et al., 2014). As a result, safe and effective non-chemical means to control *B. odoriphaga* are greatly needed.

Previous studies have shown that *B. odoriphaga* uses host plant volatiles to find hosts, mates, and oviposition sites (Yang et al., 2019). Li H. J. et al. (2007) found that *B. odoriphaga* females release n-heptadecane to attract males. Because the larvae have limited dispersal abilities, they rely on the adults to select suitable host plants for larval feeding, growth, and development. Although olfactory proteins are clearly important for regulating the behaviors of *B. odoriphaga* adults, most research concerning the functions of such proteins has focused on odorant binding proteins (BodoOBPs) (Tang et al., 2019; Yang et al., 2021a; Yang et al., 2021b) rather than on BodoCSPs.

To date, five *BodoCSP* genes have been identified from the *B. odoriphaga* antennae transcriptome (Zhao et al., 2018), but only the function of *BodoCSP1* has been studied (Zhang et al., 2021). As determined by the latter authors, *BodoCSP1* is highly expressed in antennae and is involved in the perception of host plant volatiles. The present work we conducted an extensive study to explore the potential functions of the *CSP* gene, *BodoCSP4*. We found that the *BodoCSP4* expression levels were highest in the heads (without antennae) of males, followed by the antennae of males. To determine the specific physiological functions of BodoCSP4, we characterized its binding affinity with Chinese chive volatiles, modeled its protein structure, and investigated its potential binding sites. We also used a Y-tube olfaction assay to measure the behavioral responses of *BodoCSP4*-silenced *B. odoriphaga* adult males to host plant volatiles. These results increase our understanding of chemo-sensation by *B. odoriphaga* and could identify new molecular targets for controlling this important pest.

## Materials and methods

### Insect rearing

A population of *B. odoriphaga* was obtained from a Chinese chive field in the ShunYi District of Beijing, in 2019. The population has been maintained and fed on fresh Chinese chives (not treated with insecticides) in a climate-controlled chamber at 25–28°C, 70–80% relative humidity (RH), and a 16:8 h light/dark photoperiod.

### RNA extraction and cDNA synthesis

Total RNA was extracted from eggs (n = 100), larvae (n = 10), pupae (n = 10), and adults (females and males that were <2 days old; n = 10 for each sex), and also from the antennae (n = 500), male heads (without antennae; n = 500), abdomens (n = 10), and carcasses (thoraxes, wings, and legs mixed; n = 10) of males and females. Extraction was performed with the Trizol kit (Invitrogen, CA, United States) according to the manufacturer’s instructions. The RNA samples were quantified using a Nanodrop ND-2000 spectrophotometer (Nanodrop, Wilmington, DE, United States), and the integrity was confirmed by 2% agarose gel electrophoresis.

### Tested ligands

N-phenyl-1-naphthylamine (1-NPN) was used as the competitive fluorescent reporter. The following volatiles were used in binding assays with BodoCSP4: alkanes (nonane, dodecane, tetradecane, hexadecane, n-heptadecane); terpenes (ocimene, ß-pinene, ß-caryophyllene, (R)-(+)-limonene, a-humulene); alcohols ((Z)-3-hexen-1-ol, 1,8-cineole, citronellol, linalool); esters (butyl levulinate, methyl phenylacetate, butyl acrylate); ketones (2-hexanone, beta-Ionone); aldehydes (decanal, valeric aldehyde, octanal, benzaldehyde, heptanal, nonanal); sulfur compounds (diallyl disulfide, methyl allyl disulfide) and others (acetophenone, carvaceol, h-11 indole). All of these compounds and 1-NPN were purchased from Sigma-Aldrich (St. Louis, MO, United States) and had 98% purity. Other background information on the ligands is provided in Supplementary Table S1.

### Identification, sequence analysis, and phylogenetic tree construction of *BodoCSP4*

Open reading frames (ORFs), the conserved domains, and N-terminal signal peptides of *B. odoriphaga* RNA were predicted using ORF Finder (http://www.ncbi.nlm.nih.gov/gorf/gorf.html), SMART software (http://smart.emblheidelberg.de/) (Letunic et al., 2015), and Signalp V5.1 (http://www.cbs.dtu.dk/services/SignalP/) (Jannick et al., 2004). The ExPASy Proteomics Server (http://cn.expasy.org/tools/pi_tool.html) was used to compute the isoelectric points and molecular weights of the deduced protein sequences. Primers used to validate the *BodoCSP4* sequences are listed in Table 1. Purified PCR products were cloned into the pEASY-T1 vector (TransGen, China) and sequenced.

**TABLE 1:** Primers used in cloning and expression of CSP4 in *B.odoriphaga*.

Primer name	Sequence (5′-3′)
For cloning CSP4 open reading frames	
BodoCSP4-Sense	AGG​TCA​TAA​CAG​TCA​CAA​TCA​CTT​A
BodoCSP4-Anti- sense	AAT​ACT​TTC​GGA​CAC​ACC​GAT​GTA​G
For tissue expression of CSP4	
BodoCSP4-Sense	GCA​CGA​AAG​AAG​GAC​GTG​AAC
BodoCSP4-Anti- sense	GGC​CGT​CGG​CTT​CGA​ATA​A
Heterologous expression of CSP4	
BodoCSP4-Sense	CAC​CCA​GGA​GTA​CAC​GAA​GAA​ATA​CGA​TAA​C
BodoCSP4-Anti- sense	TTA​TAG​GAA​GGA​TGA​CCT​TTT​GTT​GA
For dsRNA synthesis	
dsBodoCSP4-Sense	GGA​TCC​TAA​TAC​GAC​TCA​CTA​TAG​GTG​TAT​GTG​CAA​CTG​TGG​CAC
dsBodoCSP4-Anti- sense	GGA​TCC​TAA​TAC​GAC​TCA​CTA​TAG​GCA​TTT​GGA​GCA​ATC​TGT​TTG
dsGFP-Sense	TAA​TAC​GAC​TCA​CTA​TAG​GGG​TGT​TCA​ATG​CTT​TTC​CCG​T
dsGFP-Anti- sense	TAA​TAC​GAC​TCA​CTA​TAG​GGC​AAT​GTT​GTG​GCG​AAT​TTT​G

We used phylogenetic analysis to compare the resulting sequences of the putative CSP4 from *B. odoriphaga* with published orthologous CSP sequences (minus the signal peptides) from three other dipterans (*Drosophila melanogaster*, *Culex quinquefasciatus*, and *Anopheles gambiae*), two hemipterans (*Adelphocoris lineolatus*, *Aphis gossypii*), one hymenopteran (*Apis mellifera*), and one lepidopteran (*Bombyx mori*). Based on the predicted amino acid sequences, we used the Multhithreaded Maxmimum Likelihood method in MEGA6.0 to construct a phylogenetic tree for each gene; this was done with 1,000 bootstrap replications (Tamura et al., 2013) and with Poisson correction of distances.

### Expression of the *BodoCSP4* gene

Reverse transcription quantitative PCR (RT-qPCR) was used to quantify *BodoCSP4* expression in different developmental stages of *B. odoriphaga* (egg, larva, pupa, and adult) and in different tissues of *B. odoriphaga* adults including male heads without antennae, female antennae, male antennae, abdomens, and carcasses (thoraxes, wings, and legs mixed). RNA was extracted according to reagent protocols. Primers designed based on cDNA sequences were used for RT-qPCR (Table 1). RPL18 and RPS15 were used as reference genes for relative expression analysis of *BodoCSP4* at different life stages, and EF1 and ACT were used as reference genes for expression analysis in different tissues (Shi et al., 2016).

### Recombinant protein expression and purification of BodoCSP4 protein

The BodoCSP4 cDNA was PCR amplified with specific primers (Table 1). The PCR products were ligated into the expression vector pBM30 according to the manufacturer’s instructions (Biomed, Beijing, China). The ligation products containing the pBM30/BodoCSP4 sequence were used to transform *Escherichia coli* BL21 (DE3) cells (TransGen Biotech) for protein expression and sequencing. Positive colonies were used for expression and purification of the recombinant BodoCSP4 protein. The results showed that, after sonication and centrifugation, the BodoCSP4 proteins were mainly expressed in insoluble bodies. Protein refolding was performed according to redox methods (Prestwich, 1993). In brief, 50 mM Tris buffer (pH 6.8) containing 0.2% Triton X-100 was used to wash the insoluble inclusion body, which was then dissolved in 6 M guanidine hydrochloride. The refolded protein was then collected and purified by Ni^2+^ ion affinity chromatography (GE-Healthcare, United States). The His-tag was removed by recombinant enterokinase (rEK) (Novagen, Beijing, China). The size and the concentration of BodoCSP4 were determined using SDS-PAGE and the BCA protein assay kit (CoWinbiotech, Beijing, China), respectively.

### Fluorescence binding assays

An F-380 fluorescence spectrophotometer (Tianjin, China) was used to measure the binding affinity of BodoCSP4 to the selected volatiles, using 1-N-phenyl-naphthylamine (1-NPN) as the fluorescent probe. 1-NPN and 33 ligands were selected for this assay based on previous studies (Li H. L. et al., 2007; Zhang et al., 2016; Yang et al., 2019) (Supplementary Table S1). Both 1-NPN and the volatile compounds were diluted in chromatographic-grade methanol to make 1 mM stock solutions. Recombinant BodoCSP4 was dissolved in 20 mM Tris-HCl (pH 7.4) and diluted to a 2 µM stock solution. The dissociation constants (Kd) between 1-NPN and the recombinant BodoCSP4 were calculated by Scatchard analysis and the ligand binding affinity (Ki) was calculated with the following equation: Ki = [IC50]/(1 + [1-NPN]/K_1- NPN_), in which IC50 is the maximum concentration at which the ligand replaces 50% of the fluorescence value of 1-NPN [1-NPN] is the free concentration of 1-NPN; and K_1-NPN_ is the dissociation constant of 1-NPN (Campanacci et al., 2003; Wei et al., 2008).

### 3D structure and molecular docking of BodoCSP4

The 3D structure of BodoCSP4 was obtained with a template of CSPMbraA6 (1N8V) and Swiss-Model software. The binding cavity of BodoCSP4 was predicted using SYBYL 7.3 software. Based on the results of the fluorescence binding assays, the ligands diallyl disulfide, methyl allyl disulfide, and n-heptadecane were selected to construct the molecular conformations by Sketch mode and were optimized using the Tripos force field and Gasteiger-Hückel charge. In addition, Surflex-Dock (SYBYL 7.3) was applied to the molecular docking modeling between the BodoCSP4 protein and three ligands.

### dsRNA synthesis and Y-tube olfactometer assays

The primer pairs *dsCSP4* and *dsGFP*, containing the T7 promoter sequence, are listed in Table 1, and the T7 RiboMAX ™ Express RNAi System was used to synthesize double stranded RNAs (dsRNAs). The dsRNAs were microinjected into 3-day-old pupae of *B. odoriphaga*, and newly eclosed male adults were selected for dsRNA extraction and behavioral assays as previously described (Yang et al., 2021b; Zhang et al., 2021). According to the fluorescence binding results, after *BodoCSP4* was silenced, a Y-tube olfactometer was used to assess the behavioral responses of *B. odoriphaga* male adults to the ligands diallyl disulfide, methyl allyl disulfide, and n-heptadecane as described in our previous report (Yang et al., 2019). In brief, a 10-μL solution of diallyl disulfide, methyl allyl disulfide, or n-heptadecane in hexane was placed on a strip (4 × 40 mm) of filter paper; after 10 s, the strip was placed at the end of one of the two Y-tube arms. As a control, a hexane-treated filter paper strip was placed at the end of the other arm. Two streams of clean air were passed through the Y-tube arms to the base, and one adult male that had been previously injected (at the pupal stage, 12 h before emergence) with *dsBodoCSP4* or *dsGFP* was placed in the base of the Y-tube. If the male adult moved at least half way up one of the Y-tube arms, the result was recorded and considered an indication of attraction or repulsion depending on which arm was selected. Each of six treatment combinations (3 host plant volatiles with controls and *dsBodoCSP4* or *dsGFP* microinjected adults) are represented by 60 replicate assays.

### Statistical analysis

All RT-qPCR experiments were analyzed using one-way ANOVA followed by Tukey-HSD tests with SPSS 20.0 software. All RT-qPCR experiments were conducted using three independent biological replicates. In each of the six Y-tube olfactometer assays (3 ligands and controls without ligand × two types of microinjected *B. odoriphaga* adult males), behavioral responses to filter paper with or without ligand were analyzed by a chi-square test (χ 2).

## Results

### Identification and phylogenetic analysis of *BodoCSP4* in *B. odoriphaga*

We identified a *CSP* gene named *BodoCSP4* (Accession number: MG544173) in a previously published *B. odoriphaga* transcriptome dataset, and we verified its identity by RT-PCR (Table 2; Fig. S1A). The full-length *BodoCSP4* sequence contains an ORF of 357 bp. The predicted amino acid sequence has the typical four-cysteine signature (Wanner et al., 2004) with a motif of C_1_-X_6-8_-C_2_-X_16-21_-C_3_-X_2_-C4 (Supplementary Figure S1B) and an 18 amino acid-signal peptide in the N terminus (Supplementary Figure S1B). BodoCSP4 has a molecular weight of 13.58 kDa and an isoelectric point of 9.42 (Table 2). In a phylogenetic tree of BodoCSP4 and other insect CSPs, BodoCSP4 was clustered with the CSPs of the dipterans *D*. *melanogaster* and *A. gambiae* (Supplementary Figure S2).

**TABLE 2:** Bioinformatics analysis of odorant-binding protein genes in *B.odoriphaga*.

Gene	Acc.No	Length of ORF	Amino acid length	Signal peptide	Full ORF	pI	Mw (kDa)
BodoCSP4	MG544173	357	118	1–18	Yes	9.42	13.58

ORF, open reading frame; pI, isoelectric point; MW, molecular weight.

### Expression profile analysis of *BodoCSP4*

**FIGURE 1:**
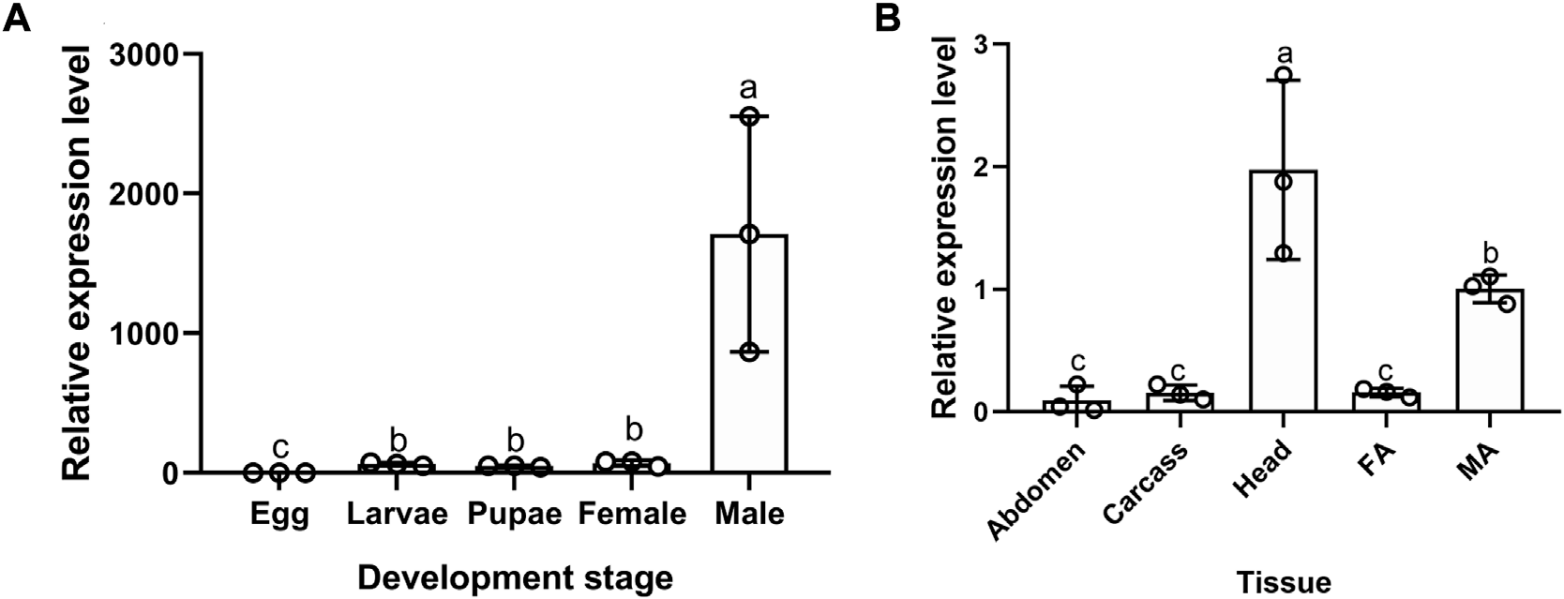
Gene expression profiling of *CSP4* in different developmental stages **(A)** and tissues **(B)** of *B. odoriphaga* as determined by RT-qPCR. Egg; Larvae; Pupae; Female; Male. Fa: Female antenna; Ma: Male antenna; Male head (without antennae); Carcass: leg + wing + thorax; Abdomen. The expression levels were estimated using the 2^−ΔΔCt^ method. The expression level in eggs was used as a standard to compare expression levels among developmental stages, and the expression level in male antennae was used as a standard to compare expression levels among tissues. Values are means ± SE; means with different letters are significantly different (*p* < 0.05).

Among developmental stages, the expression level of *BodoCSP4* was lowest in eggs and highest in adult males (F = 11.711, *df* = 4,14, *p =* 0.001) (Figure 1A). Regarding tissue-specific expression analysis in adults, expression levels of *BodoCSP4* were highest in adult male heads (antennae removed), followed by adult male antennae (F = 14.68, *df* = 4,14, *p =* 0.000) (Figure 1B).

### Recombinant protein expression and purification of BodoCSP4

The recombinant BodoCSP4 protein was successfully expressed and purified in *Escherichia coli,* yielding a high concentration (up to 0.68 mg/ml). BodoCSP4 was mainly expressed in insoluble bodies and its molecular weight is < 15 kDa, which was consistent with the predicted results (Supplementary Figure S3).

### Fluorescence binding assays with BodoCSP4

**FIGURE 2:**
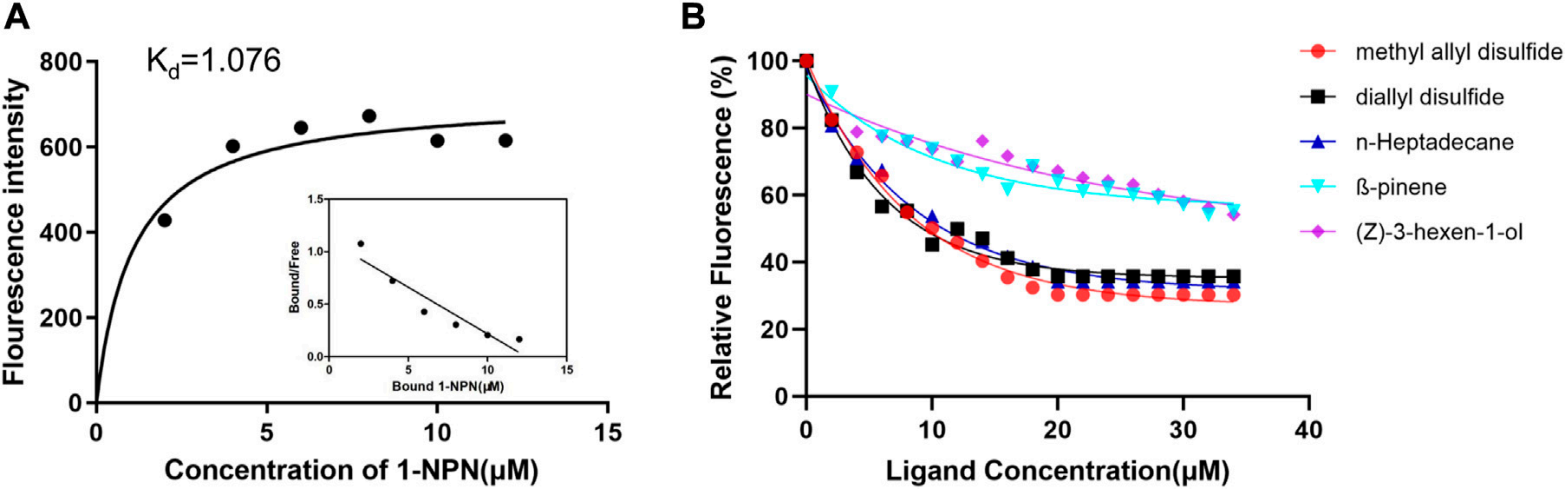
Binding curves for 1-NPN and Scatchard plots of recombinant BodoCSP4 **(A)** and fluorescence competitive binding curves of the recombinant protein BodoCSP4 with five ligands **(B)**.

To determine the function of the recombinant protein, we measured the ligand-binding affinity of recombinant CSP4 in a competitive fluorescence binding assay with 33 ligands (Figure 2 and Supplementary Table S2). The dissociation constant (K_d_) of BodoCSP4/1-NPN was 1.076 µM (Figure 2A). The binding affinity results showed that BodoCSP4 specifically bound to 3 of the 33 ligands: diallyl disulfide (5.71 µM), methyl allyl disulfide (5.71 µM), and n-heptadecane (6.85 µM) (Figure 2B). The binding affinities between BodoCSP4 and the other ligands are listed in Supplementary Table S2.

### 3D model structuring and molecular docking of BodoCSP4

**FIGURE 3:**
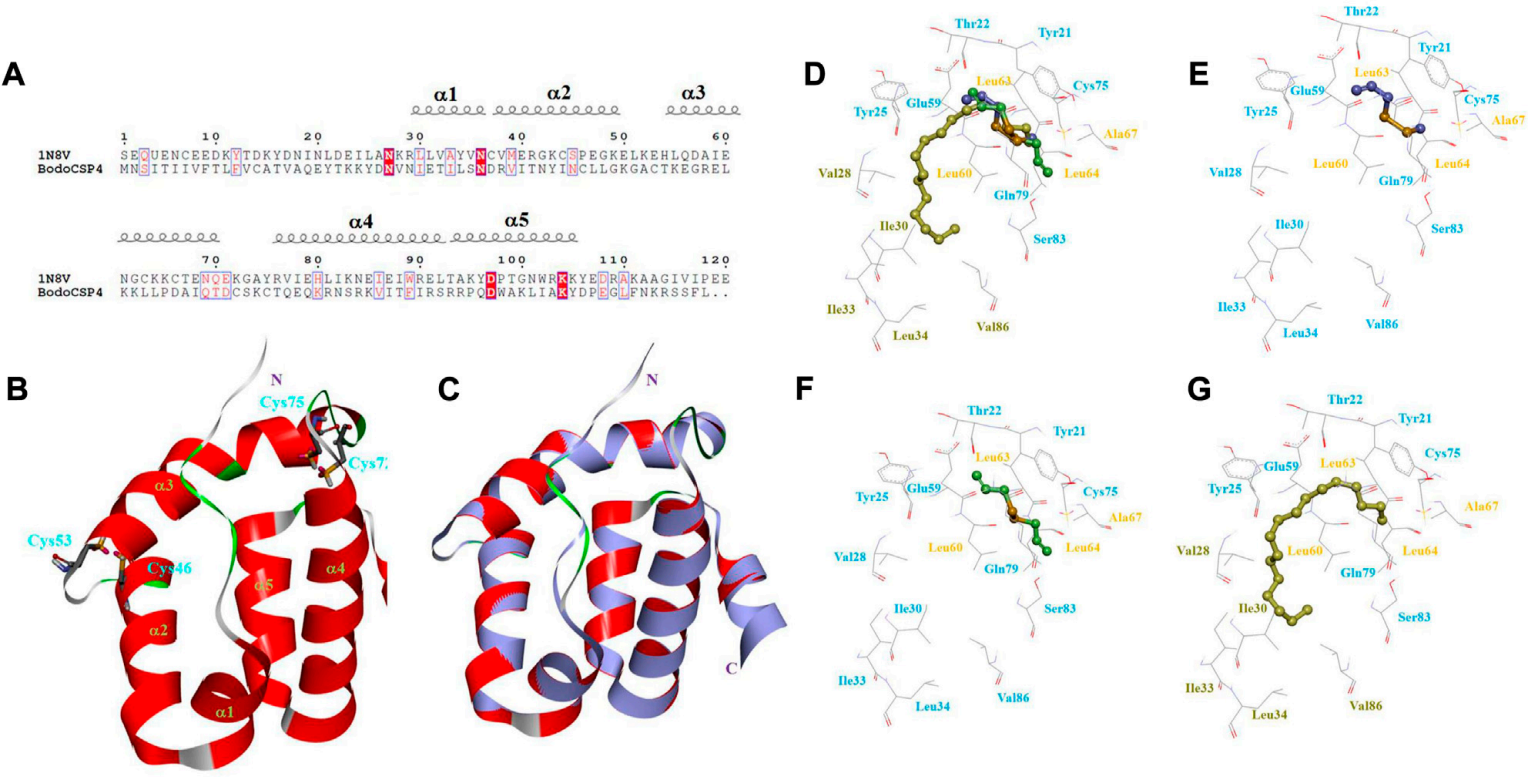
3D structure model and binding cavity of Bradysia odoriphaga chemosensory protein 4 (BodoCSP4). **(A)** Sequence alignment of BodoCSP4 and CSPMbraA6 (1N8V). The black spiral coils represent α-helices. **(B)** 3D model of the target protein BodoCSP4 based on the crystal structure of the template protein of CSPMbraA6; two disulfide bridges are labeled in blue, and the five α-helices are marked in green. **(C)** Alignment of the target protein BodoCSP4 (red) and the template protein CSPMbraA6 (purple, 1N8V). 3D models of interactions of the recombinant protein BodoCSP4 with **(D)** three ligands (diallyl disulfide, methyl allyl disulfide, and n-heptadecane), **(E)** diallyl disulfide, **(F)** methyl allyl disulfide, and **(G)** and n-heptadecane. In D-G, the yellow residues present hydrophobic residues, the blue residues represent hydrophilic residues, and the brown residues represent the hydrophobic residues of the long chain of n-heptadecane.

Based on the high alignment and high sequence identity (42%) between BodoCSP4 and the template protein CSPMbraA6 (Figure 3A), BodoCSP4 has five α-helices located between residues Asn29-Asn36 (α1), Asp37-Lys50 (α2), Thr54-Thr70 (α3), Cys75-Arg93 (α4), and Arg94-Asp106 (α5) (Figure 3B).

To better understand the potential key amino acid residues involved in the interaction between the BodoCSP4 protein and a ligand, we examined BodoCSP4 and ligand complexes by molecular docking (Figure 3). Some hydrophobic residues of BodoCSP4 surround the ligands (diallyl disulfide, methyl allyl disulfide, and n-heptadecane), including Leu60, Leu63, Leu64, and Ala67 (Figure 3C). Because diallyl disulfide, methyl allyl disulfide, and n-heptadecane have similar structures and conformations (diallyl disulfide and methyl allyl disulfide in particular have the same disulfide bond group), they have almost the same BodoCSP4 binding site (Figures 3D, E). Unlike diallyl disulfide and methyl allyl disulfide, however, n-heptadecane occupied a narrow and hydrophobic tunnel of *BodoCSP4* owing to the ligand’s long hydrophobic tail; the hydrophobic tunnel included residues Val28, Ile30, Ile33, Leu34, Val86, among others (Figure 3F). The following hydrophobic residues of BodoCSP4 surround the three ligands and are likely binding sites: Leu60, Leu63, Leu64, Ala67, Val28, Ile30, Ile33, Leu34, and Val86.

### Behavioral effect of *BodoCSP4* knockdown by RNAi

**FIGURE 4:**
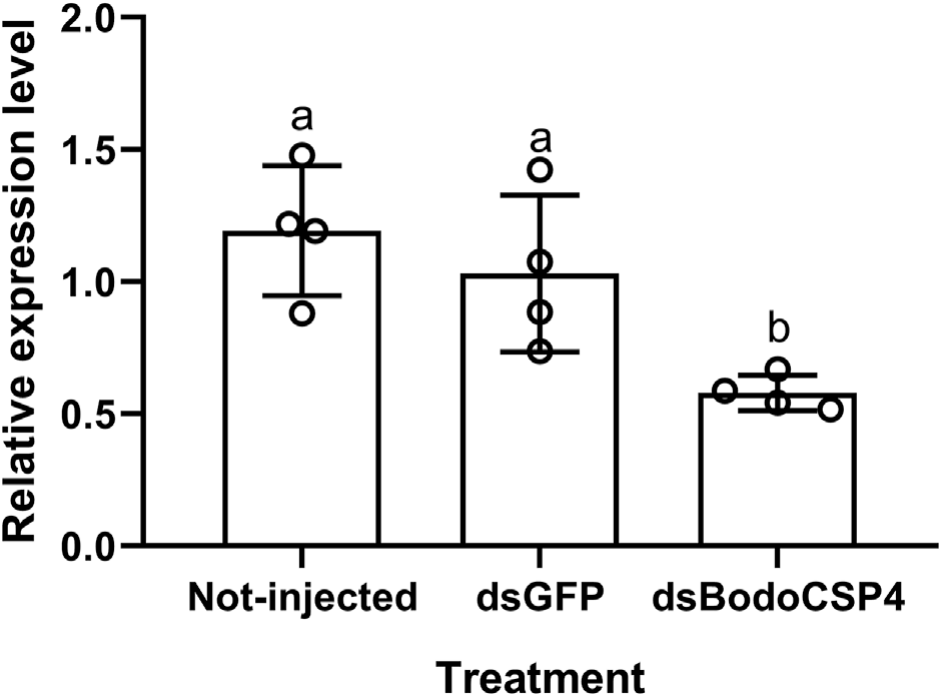
Effects of treatment of *Bradysia odoriphaga* male adults with *dsGFP* or *dsBodoCSP4* on their mRNA levels of *BodoCSP4* (as determined by RT-qPCR). The values are means ± SEM; means with different letters are significantly different (*p* < 0.05).

**FIGURE 5:**
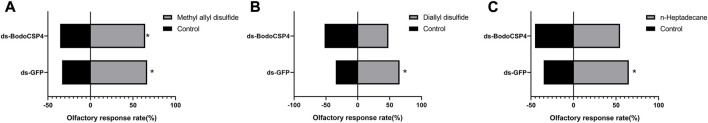
Behavioral responses of *Bradysia odoriphaga* male adults previously injected with dsRNAi to **(A)** diallyl disulfide (or a control), **(B)** methyl allyl disulfide (or a control), or **(C)** and n-heptadecane (or a control) in Y-tube olfactometer assays. In **(A–C)**, one arm of the olfactometer contained the indicated ligand in solvent (hexane), and the other arm contained the solvent alone (control). For each assay, a treated male adult was placed at the base of the olfactometer, and its movement into one of the two arms was recorded; an asterisk indicates that significantly more adults responded to the ligand than to the control in the indicated assay. For each combination of ligand/control and *dsGFP*-treated or *dsBodoCSP4*-treated adult in **(A–C)**, n = 60. Asterisks indicate statistically significant differences between the behavioral response to the ligand vs. the control as determined by chi-square tests: *p < 0.05, **p < 0.01.

To further determine whether *BodoCSP4* is important for recognizing the above three ligands, we silenced the *BodoCSP4* gene and measured the attraction of *dsBodoCSP4*- and *dsGFP*-treated adult males to each ligand or to a control without ligand in Y-tube olfaction assays. Treatment of adult males with *dsBodoCSP4,* but not with *dsGFP*, significantly reduced *BodoCSP4* expression (F = 7.952,*df* = 2,11,*p* = 0.01) (Figure 4). The *dsGFP*-treated adults were attracted to methyl allyl disulfide (Figure 5A; *χ2* = 5.952, *df* = 1, *p* = 0.025), diallyl disulfide (Figure 5B; *χ2* = 4.849, *df* = 1, *p* = 0.028), and n-heptadecane (Figure 5C; *χ2* = 4.604, *df* = 1, *p* = 0.032). The *dsBodoCSP4*-treated adults were attracted to methyl allyl disulfide (Figure 5A; *χ2* = 3.998, *df* = 1, *p* = 0.046), however no olfactory attraction to diallyl disulfide (Figure 5B; *χ2* = 0.08, *df* = 1, *p* = 0.777) and n-heptadecane (Figure 5C; *χ2* = 0.501, *df* = 1, *p* = 0.479) was observed. These results suggest that *BodoCSP4* is important for the behavioral response of *B. odoriphaga* adults to diallyl disulfide and n-heptadecane but not to methyl allyl disulfide.

## Discussion

Like odorant-binding proteins (OBPs), CSPs are important components of insect olfactory systems, and are required by insects to search for and detect host plants, suitable oviposition sites, and mates (Vogt et al., 1985; Field et al., 2000; Sun et al., 2014). Previous research clarified the physiology and function of *BodoCSP1*, but the physiology and function of other *CSPs* in *B. odoriphaga* remained unknown prior to the current study.

That CSPs are widely distributed on insect chemosensory and non-chemosensory organs suggests that they have diverse functions (Jacquin-Joly et al., 2001). The current study with *B. odoriphaga* revealed that *BodoCSP4* is mainly expressed in the heads (without antennae) of adult males, followed by the antennae of adult males. The broad expression profile of *BodoCSP4* among *B. odoriphaga* tissues indicates that *CSP4* may have other functions besides olfaction in this insect. Previous studies showed that OBP genes were highly expressed in insect heads, suggesting that they contribute to the detection of host plant volatiles and sex pheromones (Chang et al., 2015; Liu et al., 2015; Wang et al., 2016). The following studies also showed that the indicated *CSPs* are highly expressed in the male antennae of the following insects: *CSP11* of *Plutella xylostella* (Fu et al., 2020), *CSP8* of *Nilaparvata lugens* (Waris et al., 2018), *CSP1* of *Cylas formicarius* (Hua et al., 2021), and *CSP3* of *Cnaphalocrocis medinalis* (Zeng et al., 2018). High expression of CSP in male antennae suggests that they may be more involved in the perception of sex pheromones than plant volatiles. Based on the current results with *B. odoriphaga*, we speculate that *BodoCSP4* is involved not only in the searching and localization of host plants by males, but also in finding females.

To explore the role of BodoCSP4, we determined the binding affinity of BodoCSP4 to 33 ligands. The results indicated that BodoCSP4 displayed high binding affinities (Ki < 10 μM) to two sulfur-containing volatiles, diallyl disulfide and methyl allyl disulfide, and to n-heptadecane. Liu et al. (2020) found that sulfur-containing secondary isothiocyanates (ITCs) affected the preference of *P. xylostella* for *Arabidopsis thaliana*; the latter study also reported that knockdown of *PxylOr35* or *PxylOr49* reduced the effect of ITCs on *P. xylostella* selection of oviposition sites, and that knockdown of both genes reduced the attraction of P. xylostella to ITCs. In addition, researchers recently reported that ITCs are not only chemical signals used by adult Scaptomyza flava to find host plants, but are also involved in the evolution of the olfactory receptor *SflaOr67bs* (Matsunaga et al., 2022). Our previous results showed that the sulfur-containing volatiles from Chinese chives, diallyl disulfide and methyl allyl disulfide, elicit strong electrophysiological responses from the antennae of both male and female *B. odoriphaga* (Yang et al., 2019). In the current study, however, the expression levels of *BodoCSP4* were highest in male heads followed by male antennae. We therefore hypothesized that diallyl disulfide and methyl allyl disulfide are chemical cues that are specifically recognized by the olfactory gene *BodoCSP4* and may help male *B. odoriphaga* adults locate Chinese chives. Interestingly, the current results indicated that BodoCSP4 also displayed a high binding affinity with n-heptadecane, which was obtained as an extract from the abdomen of *B. odoriphaga* females and elicits a behavioral response in male adults (Li H. J. et al., 2007). This finding is similar to that for *CSPA6* of *Mamestra brassicae* (Jacquin-Joly et al., 2001) and *SinfCSP19* of *Sesamia inferen* (Zhang et al., 2014), suggesting that *BodoCSP4* may be involved in the recognition of sex pheromones by *B. odoriphaga*.

Homology modeling and molecular docking have been used to explore specific ligand-binding features (Liu et al., 2019). In the previous study, the modeled BodoCSP4 only had five α-helixes, which is similar to SfurCSP5 (Chen et al., 2018). BodoCSP4, however, differs from other insect CSPs, which contain six α-helixes (Campanacci et al., 2003; Tomaselli et al., 2006; Jean-François, 2014); as a result, it will be necessary to confirm or modify the actual 3D structure of BodoCSP4 in future studies. Kulmuni and Havukainen (2013) found that insect CSPs with five α-helixes are mostly involved in searching for mates. We therefore suggest that BodoCSP4 may be involved in finding females and in other olfactory functions.

Binding models in the current study showed that BodoCSP4 bound to the ligands of methyl allyl disulfide, diallyl disulfide, and n-heptadecane near many BodoCSP4 hydrophobic residues (including Leu60, Leu63, Leu64, and Ala67, Val28, Ile30, Ile33, Leu34, and Val86) but not the most polar hydrophobic residues (Figure 3). Mármol et al. (2021) reported that interactions with hydrophobic residues contribute to odor molecule binding and thereby mediate ligand recognition. In addition, previous studies have reported that leucine is the key amino acid for ligand binding, as was observed for *Mamestra brassicae* CSP6 (Campanacci et al., 2003), SfurCSP5 (Chen et al., 2018), and BminCSP3 (Cui et al., 2022). The hydrophobic residues of BodoCSP4 interacted with all three ligands, indicating their key involvement in ligand binding of BodoCSP4. This feature is consistent with other OBPs and CSPs (Tomaselli et al., 2006; Zheng et al., 2015; Northey et al., 2016). However, molecular docking can only assist in predicting binding mechanisms, and as such we will use site-directed mutagenesis to further investigate the specific functions of these hydrophobic amino acids. Based on both the homology modeling, molecular docking, and competitive binding results, we conclude that CSP4 can bind to both sulfur volatiles and sex pheromones, and that CSP4 therefore participates in multiple physiological functions in *B. odoriphaga*.

In the present study we used RNA interference and behavioral assays to further confirm the physiological functions of *BodoCSP4* in *B. odoriphaga*. The results indicated that silencing of the *BodoCSP4* gene significantly decreased the behavioral response of *B. odoriphaga* male adults to diallyl disulfide and n-heptadecane but did not decrease the behavioral response to methyl allyl disulfide. In contrast, Zhang et al. (2021) reported that silencing of *BodoCSP1* reduced the behavioral response of *B. odoriphaga* male adults to diallyl disulfide and methyl allyl disulfide. In addition, previous reports indicated that *BodoOBP1*, *BodoOBP2* (Tang et al., 2019), *BodoOBP5* (Yang et al., 2021a), and *BodoOBP8* (Yang et al., 2021b) bind well to diallyl disulfide and methyl allyl disulfide. We suggest that *BodoCSP4*, however, may work together with other *OBPs* or *CSPs* to recognize methyl allyl disulfide methyl allyl disulfide. For example, Sun et al. (2016) found that *CmedOBP2* and *CmedOBP3* co-regulate odorant recognition by *C. medinalis* adults, and Maguire et al. (2022) found that *AgamOR2* and *ORCO* can together alter the olfactory preferences of *Anopheles mosquitoes*. Based on the current results, we speculate that diallyl disulfide and n-heptadecane but not methyl allyl disulfide can be specifically recognized by *BodoCSP4*, and that diallyl disulfide and n-heptadecane are the main chemical signals perceived by *BodoCSP4* in host plant recognition and localization by males, as well as female localization.

Overall, the results of this study suggest that *BodoCSP4* may be involved in the recognition and localization of host plants and mates. Determining whether diallyl disulfide and n-heptadecane function as attractants to regulate *B. odoriphaga* behavior will require additional research.

## Data Availability

The original contributions presented in the study are included in the article/Supplementary Materials, further inquiries can be directed to the corresponding author.
