# Supplementary material for: Chemosensory protein 4 is required for Bradysia odoriphaga to be olfactory attracted to sulfur compounds released from Chinese chives
Source: Front Physiol. 2022 Sep 27;13:989601. doi: 10.3389/fphys.2022.989601 (PMC9552003; doi:10.3389/fphys.2022.989601)
Supplement: Supplementary file 1 [file DataSheet1.docx]

**Supplemental Table1:** Chemical compound in the binding assays of recombinant protein BodoCSP4.

| Chemical compound | Molecular | Formula | Pourity | Source | CAS |
| --- | --- | --- | --- | --- | --- |
| Nonane | 128.26 | C_9_H_20_ | 98% | Sigma-Aldrich | 111-84-2 |
| Dodecane | 170.33 | CH_3_(CH_2_)_10_CH_3_ | 98% | Sigma-Aldrich | [112-40-3](https://www.chemicalbook.com/ProductChemicalPropertiesCB5678167.htm) |
| Tetradecane | 198.39 | C_14_H_30_ | 98% | Sigma-Aldrich | 629-59-4 |
| Hexadecane | 226.44 | C_16_H_34_ | 98% | Sigma-Aldrich | 544-76-3 |
| n-Heptadecane | 240.47 | C_17_H_36_ | 98% | Sigma-Aldrich | 629-78-7 |
| Ocimene | 136.24 | C_10_H_16_ | 98% | Sigma-Aldrich | 3016-19-1 |
| ß-pinene | 136.23 | C_10_H_16_ | 98% | Sigma-Aldrich | 127-91-3 |
| ß-caryophyllene | 204.35 | C_15_H_24_ | 98% | Sigma-Aldrich | 87-44-5 |
| (R)-(+)-Limonene | 136.23 | C_10_H16 | 98% | Sigma-Aldrich | 5989-27-5 |
| a-Humulene | 204.36 | C_15_H_24_ | 98% | Sigma-Aldrich | 6753-98-6 |
| （Z）-3-Hexen-1-ol | 100.16 | C_6_H_12_O | 98% | Sigma-Aldrich | 928-96-1 |
| 1,8-Cineole | 154.25 | C_10_H_18_O | 98% | Sigma-Aldrich | 470-82-6 |
| Citronellol | 156.26 | C_10_H_20_O | 98% | Sigma-Aldrich | 106-22-9 |
| Linalool | 154.25 | C_10_H_18_O | 98% | Sigma-Aldrich | 78-70-6 |
| Butyl levulinate | 172.22 | C_9_H_16_O_3_ | 98% | Sigma-Aldrich | 2052-15-5 |
| Methyl phenylacetate | 150.17 | C_9_H_10_O_2_ | 98% | Sigma-Aldrich | 101-41-7 |
| Butyl Acrylate | 128.17 | C_7_H1_2_O_2_ | 98% | Sigma-Aldrich | 119-36-8 |
| 2-Hexanone | 100.16 | C_6_H_12_O | 98% | Sigma-Aldrich | 591-78-6 |
| Beta-Ionone | 192.30 | C_13_H_20_O | 98% | Sigma-Aldrich | 14901-07-6 |
| Decanal | 156.26 | C_10_H_20_O | 98% | Sigma-Aldrich | 112-31-2 |
| Valeric aldehyde | 86.13 | C_5_H_10_O | 98% | Sigma-Aldrich | 110-62-3 |
| Octanal | 128.21 | C_8_H_16_O | 98% | Sigma-Aldrich | 124-13-0 |
| benzaldehyde | 106.12 | C_6_H_5_CHO | 98% | Sigma-Aldrich | 100-52-7 |
| Dodecyl aldehyde | 184.32 | C_12_H_24_O | 98% | Sigma-Aldrich | 112-54-9 |
| heptanal | 114.19 | C_7_H_14_O | 98% | Sigma-Aldrich | 111-71-7 |
| nonanal | 142.24 | C_9_H_18_O | 98% | Sigma-Aldrich | 124-19-6 |
| Diallyl disulfide | 162.26 | C_6_H_10_OS_2_ | 98% | Sigma-Aldrich | [2179-57-9](https://www.chemicalbook.com/ProductChemicalPropertiesCB3337107.htm) |
| Methyl allyl disulfide | 120.24 | C_4_H_8_S_2_ | 98% | Sigma-Aldrich | 2179-58-0 |
| Acetophenone | 120.14 | C_8_H_8_O | 98% | Sigma-Aldrich | 98-86-2 |
| Carvaceol | 150.22 | C_10_H_14_O | 98% | Sigma-Aldrich | 499-75-2 |
| （H-11）indole | 117.15 | C_8_H_7_N | 98% | Sigma-Aldrich | 120-72-9 |

**Supplemental Table 2:** Analysis of binding properties of BodoCSP4 with thirty-three compounds

| Chemical compound | BodoCSP4 | |
| --- | --- | --- |
|  | Ki(μM) | IC50(μM) |
| Nonane | >50 | >50 |
| Dodecane | >50 | >50 |
| Tetradecane | >50 | >50 |
| Hexadecane | >50 | >50 |
| Ocimene | >50 | >50 |
| ß-pinene | >50 | >50 |
| ß-caryophyllene | >50 | >50 |
| (R)-(+)-Limonene | >50 | >50 |
| a-Humulene | >50 | >50 |
| cis-3-Hexen-1-ol | >50 | >50 |
| 1,8-Cineole | >50 | >50 |
| Citronellol | >50 | >50 |
| Linalool | >50 | >50 |
| Butyl levulinate | >50 | >50 |
| Methyl phenylacetate | >50 | >50 |
| Butyl Acrylate | >50 | >50 |
| 2-Hexanone | >50 | >50 |
| Beta-Ionone | >50 | >50 |
| Decanal | >50 | >50 |
| Valeric aldehyde | >50 | >50 |
| Octanal | >50 | >50 |
| benzaldehyde | >50 | >50 |
| Dodecyl aldehyde | >50 | >50 |
| heptanal | >50 | >50 |
| nonanal | >50 | >50 |
| Diallyl disulfide | 10 | 5.71 |
| Methyl allyl disulfide | 10 | 5.71 |
| Acetophenone | >50 | >50 |
| Carvaceol | >50 | >50 |
| （H-11）indole | >50 | >50 |
| n-Heptadecane | 12 | 6.85 |
